# NRF1 and ZSCAN10 bind to the promoter region of the SIX1 gene and their effects body measurements in Qinchuan cattle

**DOI:** 10.1038/s41598-017-08384-1

**Published:** 2017-08-11

**Authors:** Da-Wei Wei, Lin-Sheng Gui, Sayed Haidar Abbas Raza, Song Zhang, Rajwali Khan, Li Wang, Hong-Fang Guo, Lin-Sen Zan

**Affiliations:** 1 College of Animal Science and Technology, Northwest A&F University, Yangling, 712100 Shaanxi People’s Republic of China; 2 National Beef Cattle Improvement Center, Northwest A&F University, Yangling, 712100 Shaanxi People’s Republic of China; 3 Modern Cattle Biotechnology and Application of National-Local Engineering Research Center, Yangling, 712100 Shaanxi People’s Republic of China; 4 Shaanxi Beef Cattle Engineering Research Center, Yangling, 712100 Shaanxi People’s Republic of China

**Keywords:** DNA methylation, Cell growth

## Abstract

The SIX1 homeobox gene belongs to the six homeodomain family and is widely thought to play a principal role in mediating of skeletal muscle development. In the present study, we determined that the bovine SIX1 gene was highly expressed in the *longissimus thoracis* and physiologically immature individuals. DNA sequencing of 428 individual Qinchuan cattle identified nine single nucleotide polymorphisms (SNPs) in the promoter region of the SIX1 gene. Using a series of 5′ deletion promoter plasmid luciferase reporter assays and 5′-rapid amplification of cDNA end analysis (RACE), two of these SNPs were found to be located in the proximal minimal promoter region −216/−28 relative to the transcriptional start site (TSS). Correlation analysis showed the combined haplotypes H_1_-H_2_ (-GG-GA-) was significantly greater in the body measurement traits (BMTs) than the others, which was consistent with the results showing that the transcriptional activity of Hap2 was higher than the others in Qinchuan cattle myoblast cells. Furthermore, the electrophoretic mobility shift assays (EMSA) and chromatin immunoprecipitation assay (ChIP) demonstrated that NRF1 and ZSCAN10 binding occurred in the promoter region of diplotypes H_1_-H_2_ to regulate SIX1 transcriptional activity. This information may be useful for molecular marker-assisted selection (MAS) in cattle breeding.

## Introduction

Body measurement traits (BMTs) are relevant indicators in cattle selection and breeding. Qinchuan cattle are known to be good beef cattle in China, because of distinctive qualities including good adaptability and fine beef flavor among others. However, these cattle also exhibit certain drawbacks, such as slow growth and underdeveloped hind hip. Accordingly, it is necessary to select important functional genes to solve these problems and increase the BMTs of beef cattle through marker-assisted selection (MAS), which is recognized as a powerful and efficient strategy compared to traditional breeding methods^1^. However, quantitative traits such as BMTs are controlled by many genes with minor effects^2^. The identification of statistically significant associations between genetic variants within candidate genes provides a potentially powerful approach to accelerate breeding efforts related to these traits for Qinchuan cattle breed improvement^3^.

The SIX homeodomain family have been identified six members to date, and designated as SIX1–SIX6^4^. The sine oculis homeobox homologue 1 (SIX1) gene is localized in both the cytoplasm and nucleus of mesenchymal stem cells during embryogenesis and plays important roles in the formation and development of various organs, such as the cranial ganglia^5^, inner ear^6^, kidney^7^, olfactory^8^ and skeletal muscle^9, 10^. During skeletal muscle development, SIX1 regulates the expression of the myogenic regulatory factors MyoD and myogenin to mediate skeletal muscle growth and regeneration^10–12^. Additionally, SIX1-null mice die at birth due to hypoplasia and an abnormal structure of primary myogenesis caused by a reduction in and delayed activation of the MyoD and myogenin genes in the limb buds^6, 12^. Moreover, SIX1 can be activated by the phosphorylation of its cofactor Eya and drives the transformation of the slow-twitch muscle phenotype towards the fast-twitch (glycolytic) phenotype^5, 13^. Additionally, the knockdown SIX1 expression causes a fibre-type shift towards a slower phenotype by affectings the MHC isoform, which is expressed in the myofibres^14^. Slow-twitch and fast-twitch muscle fibres are the two most important factors regulating meat tenderness^15^. Altogether, SIX1 is critical for embryonic development, particularly for skeletal muscle.

Despite its clear role in regulating the formation of muscle and other tissues, the mechanism by which the bovine SIX1 gene is associated with BMTs in cattle has not been reported to date. Promoter region variants can influence transcription activity by altering the transcription factor binding sites, thereby affecting individual development^16^. Herein, the objectives of this study were to identify the genetic polymorphisms at the 5′UTR of the bovine SIX1 gene and confirm potential *cis*-acting elements that are associated with single nucleotide polymorphisms (SNPs) and BMTs in Qinchuan cattle.

## Results

### Detection of SIX1 expression in bovine tissues and organs

**Figure 1:**
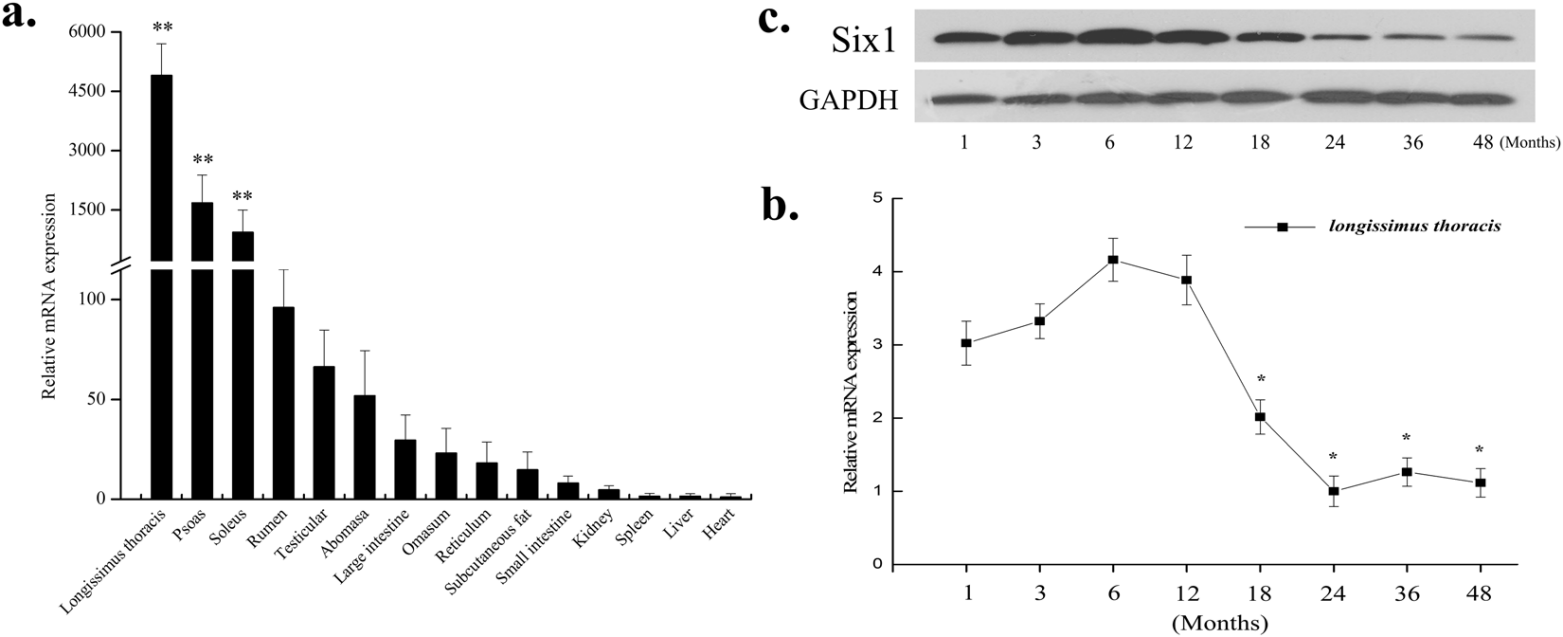
Expression pattern analysis of the bovine SIX1. (**a**) Analysis of the bovine SIX1 expression pattern in tissues and organs. (**b**) Expression pattern of the bovine SIX1 mRNA at different developmental stages. (**c**) Bovine Six1 expression pattern at different developmental stages. The samples of the *longissimus thoracis* were obtained at 1, 3, 6, 12, 18, 24, 36 and 48 months after birth. SIX1 mRNA expression was normalized to the housekeeping gene GAPDH and the expression levels were calculated relative to the gene expression in the liver and 24 months, respectively. The value of each column represents the mean ± standard deviation of three independent experiments. The unpaired Student’s t-test was used to detect significant differences. “*”*P* < 0.05 and “**”*P* < 0.01.

The distribution of bovine SIX1 mRNA was determined through qPCR using cDNA from the following 15 bovine tissues and organs: heart, liver, spleen, kidney, rumen, reticulum, omasum, abomasa, small intestine, large intestine, subcutaneous fat, *longissimus thoracis*, soleus, psoas and testicular tissue. (Fig. 1a). SIX1 had a broad tissue distribution in the cattle tissues and organs. However, the bovine SIX1 gene was highly expressed in the *longissimus thoracis*, psoas and soleus, moderately expressed in the rumen, testicular and abomasa, and only slightly expressed in the large intestine, omasum, reticulum, subcutaneous fat, small intestine, kidney, spleen, liver and heart tissue.

### Temporal expression of SIX1 in *longissimus thoracis* during different developmental stages

The expression of the bovine SIX1 gene in ***longissimus thoracis*** at 1, 3, 6 and 12 months of age was significantly higher than that during any other stages (Fig. 1b and c). However, the expression level was reduced dramatically after 18 months (Fig. 1b and c), but no significant difference was observed in the expression level after 24 months, and the expression level trend remained stable at both the mRNA and protein levels. Based on these results, there appears to be a close relationship between the growth rate traits and SIX1 gene expression in Qinchuan cattle.

### Determination of the transcription start site of the SIX1 gene

**Figure 2:**
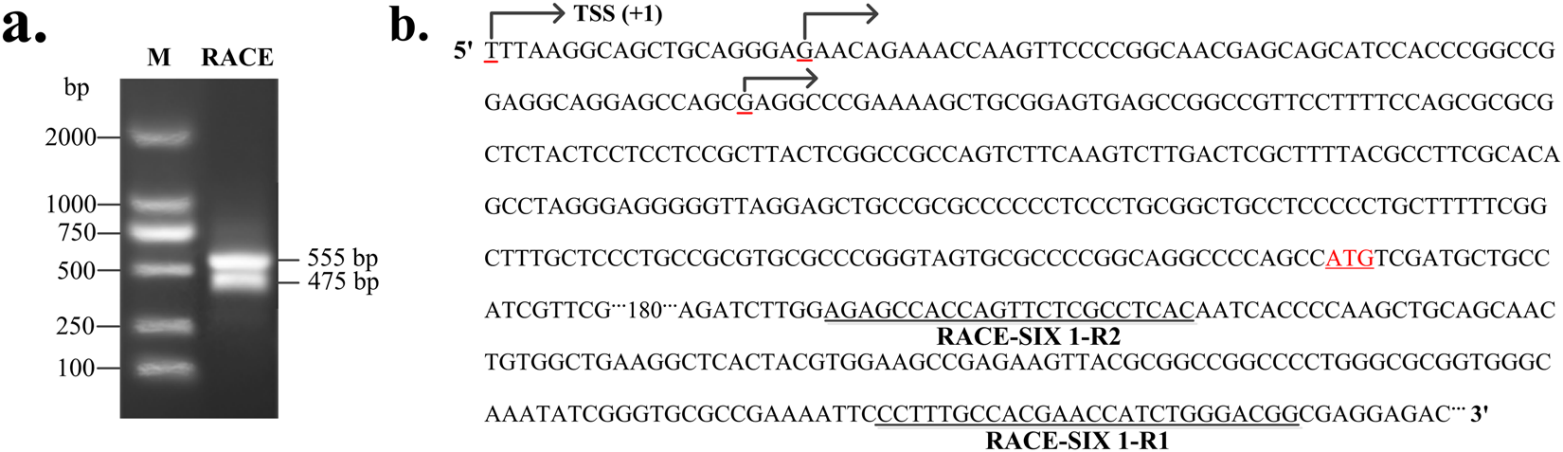
Results of 5′RACE analysis of the SIX1 cDNA from skeletal muscle. (**a**) Products of the 5′ RACE of the SIX1 (arrow) from nested PCR were analysed by agarose gel electrophoresis. (**b**) Sequence of the SIX1 mRNA region. The primers (R1 and R2) were used for the 5′ RACE analysis are underlined. The positions of identified TSS are marked with arrows and red underline. The translational start site (ATG) is shown in red letters.

To analyze the structure and molecular mechanisms of the bovine SIX1 gene, we performed 5′-RACE to identify the transcriptional start site (TSS) of SIX1. Two successive rounds of PCR were performed using antisense primer R1 and nested primer R2 (Table S1). As shown in Fig. 2a, two bands of 555 and 475 bp were amplified. In total, 18 positive clones had three different 5′ ends in the first exons, i.e., at 316, 297 and 236 bp upstream of the TSS (Fig. 2b). Sequence alignment showed that the most upstream of the TSS was completely in accord with the published SIX1 mRNA sequence (XM_588692.7). Therefore, we verified the site and designated as +1.

### Seven SNPs were identified in the 1.8 kb upstream region of the bovine SIX1 gene

**Figure 3:**
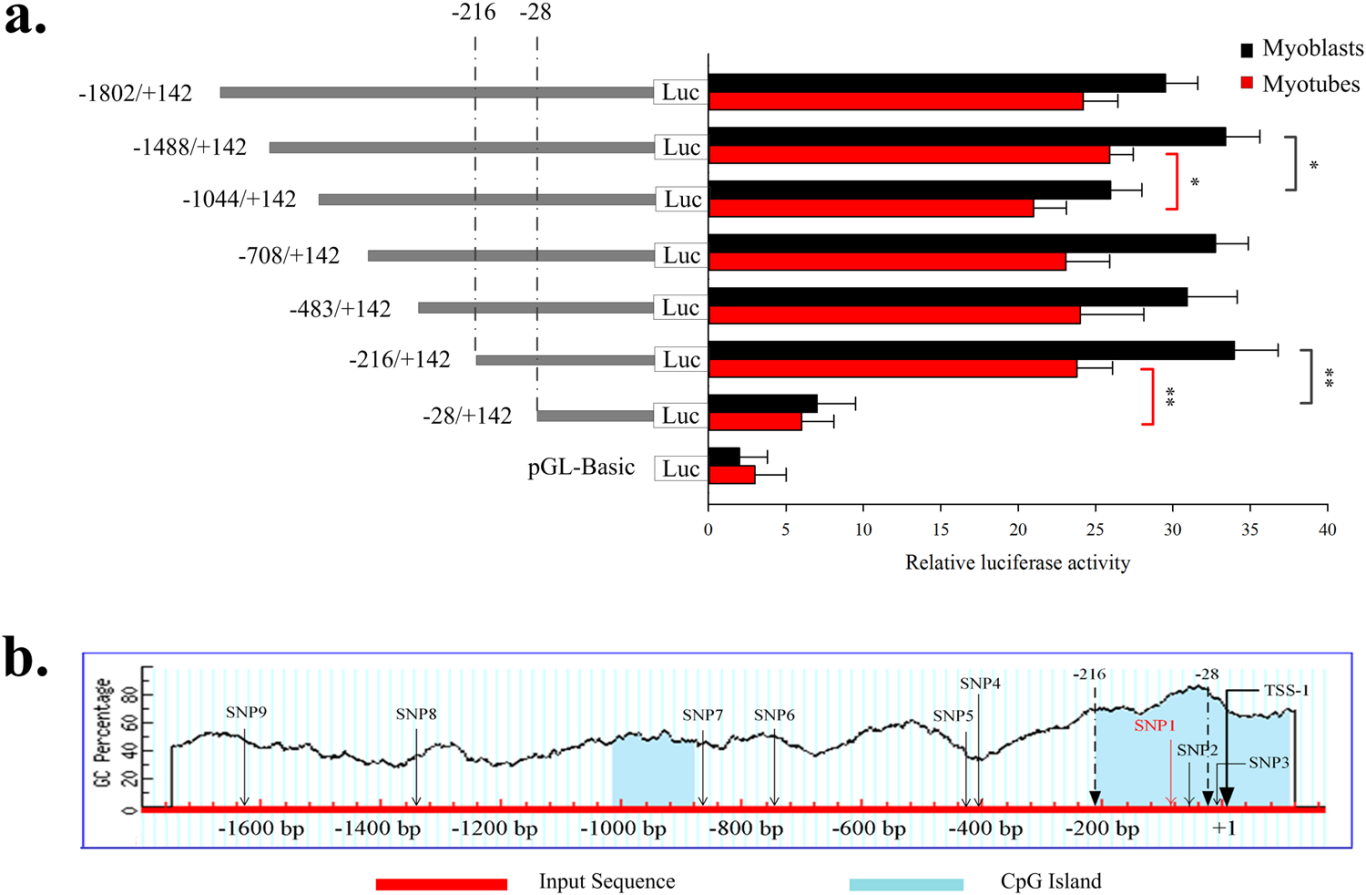
Luciferase activities in the bovine SIX1 promoter constructs in QCMCs. (**a**) All plasmids containing 5′ unidirectional deletions of the promoter region of the SIX1 gene (pGL3 −1802, −1488, −1044, −708, −483, −216, −28 and pGL3-Basic) were transfected into QCMCs. After 5 h we replaced the transfection mixture with DMEM with 2% HS (myotubes). The cells were collected for the luciferase assay at 48 h. The results are expressed as the mean ± standard deviation in arbitrary units based on firefly luciferase activity normalized to the Renilla luciferase activity in triplicate transfections. The unpaired Student’s t-test was used to detect significant differences. “*”*P* < 0.05 and “**”*P* < 0.01. (**b**) A graphical representation of the bovine SIX1 gene proximal promoter region from +1 to −1802 base pairs, predicting the regions with a high GC content. Folded lines indicate the GC percentage, which is represented on the y-axis, and the x-axis denotes the bp position on the 5′ untranslated region; the bottom of the blue area indicates the relative positions of the CpG islands. Coordinates are given relative to the translational start site (shown as +1). Arrows indicate TSS-1, positions −216 and −28 bp and nine SNP loci in the promoter, of which SNP1 and SNP2 were located in the proximal minimal promoter of the SIX1 gene.

The bovine SIX1 gene located on chromosome 10, contains two exons and two introns and encodes a protein of 284 amino acids. In this study, nine SNPs were identified in the 5′ region of the bovine SIX1 gene by DNA pool sequencing [AC_000167.1: g. −1633 G > A (SNP 9); −1357G > T (SNP 8); −867 G > A (SNP 7); −742 T > C (SNP 6); −421 C > T (SNP 5); −409 T > C (SNP 4); −85 G > T (SNP1); g. −63 G > A (SNP1); g. −14 A > G (SNP 3)] (Fig. 3b).

### Isolation of the functional proximal minimal promoter of the SIX1 gene

The proximal minimal promoter of the SIX1 gene was isolated to determine the SNPs that are responsible for influencing the function of promoter. Seven reporter constructs with progressive deletions from the 5′ end of the promoter were generated. The luciferase reporter constructs, named pGL3-1802, pGL3-1488, pGL3-1044, pGL3-708, pGL3-483, pGL3-216 and pGL3-28, were transfected into Qinchuan cattle myoblast cells (QCMCs). The luciferase assays revealed a 5-7-fold increase in the promoter activity of pGL-1802/+142 compared to that in the empty vector, indicating a functional promoter in the −1802/+142 region of the SIX1 gene. When the promoter was deleted to position −1044, the promoter activity of pGL-1044/+142 was decreased by 32% compared with the pGL-1488/+142 (Fig. 3a). After inducing the QCMCs via HS after the transfection, the results of the luciferase assays were similar to those during the undifferentiated stage (Fig. 3a). This result demonstrated that positive regulatory elements were located in the −1488/−1044 region. However, when the promoter was further deleted to −28 bp, the promoter activity of pGL-28/+142 was almost abolished in both the undifferentiated and differentiated QCMCs compared to that of pGL-216/+142. These results indicate that the proximal minimal promoter of the SIX1 gene is located within the region −216/−28 relative to TSS-1 and that the undifferentiated cell model is better for determining transcriptional activity.

### Genetic polymorphism of in the Qinchuan Cattle SIX1 proximal minimal promoter region

In the present study, the genetic parameters of the bovine SIX1 gene, including the genotype and allele frequencies, were directly calculated for all 428 animals. SNP1 (g. −85 G > T) and SNP2 (g. −63 G > A) were located in the proximal minimal promoter region −216/−28 of the SIX1 gene. We also determined the genotypic and allelic frequencies, genetic diversity parameters of heterozygosity (*He*), effective allele numbers (*Ne*), polymorphism information content (*PIC*) and Hardy-Weinberg equilibrium (Table 1). The results indicated that for the g. −85 G > T mutation, the TT genotype (14.96%) was less frequent than the wild allele GG (44.16%). The allele frequencies, *He*, *Ne* and *PIC* at the current locus were 0.6460 (G), 0.3540 (T), 0.4574, 1.8428 and 0.3528, respectively. For the g. −63 G > A mutation, the GG genotype was the most prevalent (62.15%) followed by GG (30.84%) and AA (7.01%). The values of the allele frequencies, *He*, *Ne*, and *PIC* at this locus were 0.7757 (G), 0.2243 (A), 0.3480, 1.5537 and 0.2874, respectively. In our study, g. −85 G > T and g. −65 G > A had an intermediate level of polymorphism (high level, *PIC* > 0.5; moderate level, 0.5 < *PIC* > 0.25; low level, *PIC* < 0.25)^17^. The genotypic distributions of SNP1 and SNP2 were in Hardy-Weinberg disequilibrium (chi-square test, χ^2^ < χ^2^_0.05_).

**Table 1:** Genotype frequencies (%) of the SIX1 gene for the SNPs in the Qinchuan cattle populations.

Locus	Genotypic Frequencies (N)			Total	Allelic Frequencies		χ^2^ (HWE)	*PIC*	*He*	*Ne*
	GG	GT	TT		G	T				
g.−85 G > T	189	175	64	428	0.6460	0.3540	4.8078	0.3528	0.4574	1.8428
	0.4416	0.4089	0.1496							
	GG	GA	AA		G	A				
g. −63 G > A	266	132	30	428	0.7757	0.2243	5.5335	0.2874	0.3480	1.5337
	0.6215	0.3084	0.0701							

HWE, Hardy-Weinberg equilibrium; χ0.05^2^ = 5.991, χ0.01^2^ = 9.210.

### Effects of single marker on BMTs

The association between the two SNPs (g. −85 G > T and g. −63 G > A) and the seven economic traits of body length (BL), withers height (WH), chest depth (CD), chest circumference (CC), back fat thickness (BF), ultrasound loin muscle area (ULA) and intramuscular fat content (IFC) are presented in Table 2. Individuals with genotype the GG had a significantly greater CC and IFC than those with the TT genotype (*P* < 0.05) for g. −85 G > T. For g. −63 G > A, individuals with the AA genotype had a significantly greater BL, CD, CC, BF and ULA than those with the GA and GG genotypes (*P* < 0.05) (Table 2). These results indicated that the two loci g. −85 G > T and g. −63 G > A were associated with the BMTs in Qinchuan cattle.

**Table 2:** Association of different genotypes of SNPs in SIX1 with body measurements in Qinchuan cattle.

Locus	Genotypes	BL (cm)	WH (cm)	CD (cm)	CC (cm)	BF (cm)	ULA (cm^2^)	IMF (%)
g. −85 G > T	GG (189)	130.877 ± 0.601	118.907 ± 0.417	57.783 ± 0.422	164.772 ± 0.963^a^	0.879 ± 0.019	45.588 ± 0.742	7.627 ± 0.051^A^
	GT (175)	132.174 ± 0.625	118.629 ± 0.433	57.971 ± 0.439	162.054 ± 1.001	0.860 ± 0.020	44.695 ± 0.771	7.644 ± 0.073^A^
	TT (63)	133.056 ± 1.041	120.722 ± 0.722	58.444 ± 0.731	160.333 ± 1.668^b^	0.878 ± 0.033	43.073 ± 1.286	6.118 ± 0.153^B^
	*P*	0.071	0.165	0.469	0.029	0.507	0.303	0.000
g. −63 G > A	GG (266)	130.164 ± 0.483^B^	118.318 ± 0.346	56.985 ± 0.793^b^	159.032 ± 0.016^Bb^	0.840 ± 0.016^b^	44.488 ± 0.627	7.621 ± 0.066
	GA (132)	133.043 ± 0.686^B^	119.697 ± 0.491	58.841 ± 0.487^b^	163.250 ± 1.126^b^	0.825 ± 0.023^b^	45.513 ± 0.889	7.527 ± 0.069
	AA (30)	139.850 ± 1.438 ^A^	122.883 ± 1.029	62.600 ± 1.022^a^	168.033 ± 1.362^Aa^	0.911 ± 0.048^a^	45.283 ± 1.866	7.247 ± 0.114
	*P*	0.000	0.058	0.013	0.005	0.028	0.374	0.062

Values are shown as the means ± standard error. Values with different superscripts in the same column differ significantly at *P* < 0.05 (a,b) and *P* < 0.01 (A,B) after Bonferroni correction. Body length (BL),withers height (WH), chest depth (CD), chest circumference (CC), back fat thickness (BF), ultrasound loin muscle area (ULA) and intramuscular fat content (IFC).

### Effects of diplotypes on BMTs

To further analyse the associations between the diplotypes of the two the SNPs and the BMTs, six diplotypes were combined by four haplotypes (Hap1, Hap2, Hap3 and Hap4) (Table 3) in this sample of Qinchuan cattle. Compared with the other diplotype results, the H_1_-H_2_ diplotypes had a significantly greater BL, CD, CC, BF, ULA and IFC (*P* < 0.05) than the H_1_-H_1_, H_1_-H_3_ and H_3_-H_3_ diplotypes (Table 4). Additionally, the H_3_-H_4_ diplotypes had a significantly higher BL, CD, CC and BF (*P* < 0.05) than the H_1_-H_1_, H_1_-H_3_ and H_3_-H_3_ diplotypes (Table 4). In contrast, the H_3_-H_4_ diplotypes displayed a reduced IFC (*P* < 0.05).

**Table 3:** Haplotypes of the bovine SIX1 gene and their frequencies in Qinchuan cattle.

**Haplotype**	**g. −85 G > T**	**g. −63 G > A**	**Frequency (%)**
Hap1	G	G	0.513
Hap2	G	A	0.133
Hap3	T	G	0.163
Hap4	T	A	0.091

The r^2^ value of g. −85 G > T and g. −63 G > A was 0.158.

**Table 4:** Associations of diplotypes with body measurements in Qinchuan cattle.

Diplotype	Body Measurement				Meat Quality Trait		
	BL (cm)	WH (cm)	CD (cm)	CC (cm)	BF (cm)	ULA (cm^2^)	IFC (%)
H_1_-H_1_ (125)	129.772 ± 0.716^B^	118.596 ± 0.504	57.268 ± 0.500^b^	159.984 ± 1.159^b^	0.850 ± 0.023^b^	41.558 ± 0.915^Bb^	7.703 ± 0.071^a^
H_1_-H_2_ (51)	136.612 ± 1.121^Aa^	119.745 ± 0.504	61.084 ± 0.783^Aa^	167.157 ± 1.815^Aa^	0.944 ± 0.036^Aa^	46.588 ± 1.433^A^	7.811 ± 0.112^a^
H_1_-H_3_ (107)	130.804 ± 0.774^b^	117.762 ± 0.545	57.140 ± 0.540^b^	159.051 ± 1.253^b^	0.844 ± 0.025^b^	44.256 ± 0.989	7.630 ± 0.077^a^
H_1_-H_4_ (54)	132.907 ± 1.090	119.407 ± 0.767	60.259 ± 0.760^Aa^	163.315 ± 1.764	0.871 ± 0.035	44.780 ± 1.392^a^	7.650 ± 0.109
H_3_-H_3_ (34)	129.588 ± 1.373^B^	119.044 ± 0.967	55.456 ± 0.958^B^	155.471 ± 1.223^B^	0.789 ± 0.044^B^	41.287 ± 1.755^Bb^	7.638 ± 0.137
H_3_-H4 (27)	136.019 ± 1.541^Aa^	120.074 ± 1.085	61.065 ± 1.075^Aa^	167.074 ± 1.495^Aa^	0.997 ± 0.050^Aa^	43.949 ± 1.969	7.319 ± 0.154^b^
P	0.005	0.081	0.000	0.006	0.007	0.001	0.038

Values are shown as the means ± standard error. Values with different superscripts in the same column differ significantly at *P* < 0.05 (a,b) and *P* < 0.01 (A,B) after Bonferroni correction. Body length (BL),withers height (WH), chest depth (CD), chest circumference (CC), back fat thickness (BF), ultrasound loin muscle area (ULA) and intramuscular fat content (IFC).

### Potential transcription-factors in the SIX1 haplotypes possessed different transcriptional activities

**Figure 4:**
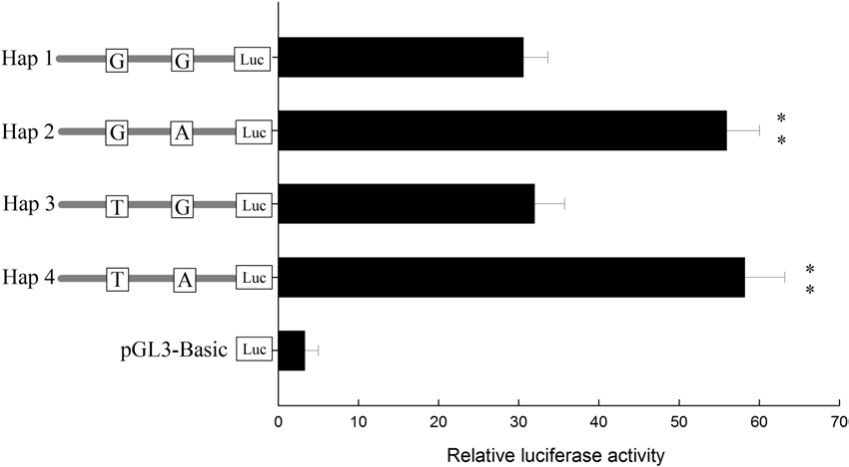
Constitutive activities of the bovine SIX1 haplotypes in QCMCs. The relative lucferase of the transcriptional activities in the candidate haplotypes was used to normalize the promoter activity. Haplotypes of the proximal minimal promoter region of the SIX1 gene were transfected into the QCMCs, and pGL3-Basic served as a control. The cells were collected for the luciferase assay at 48 h. The results are expressed as the mean ± standard deviation in arbitrary units based on the firefly luciferase activity normalized to the renilla luciferase activity in triplicate transfections. The unpaired Student’s t-test was used to detect significant differences. “*”*P* < 0.05 and “**”*P* < 0.01.

**Figure 5:**
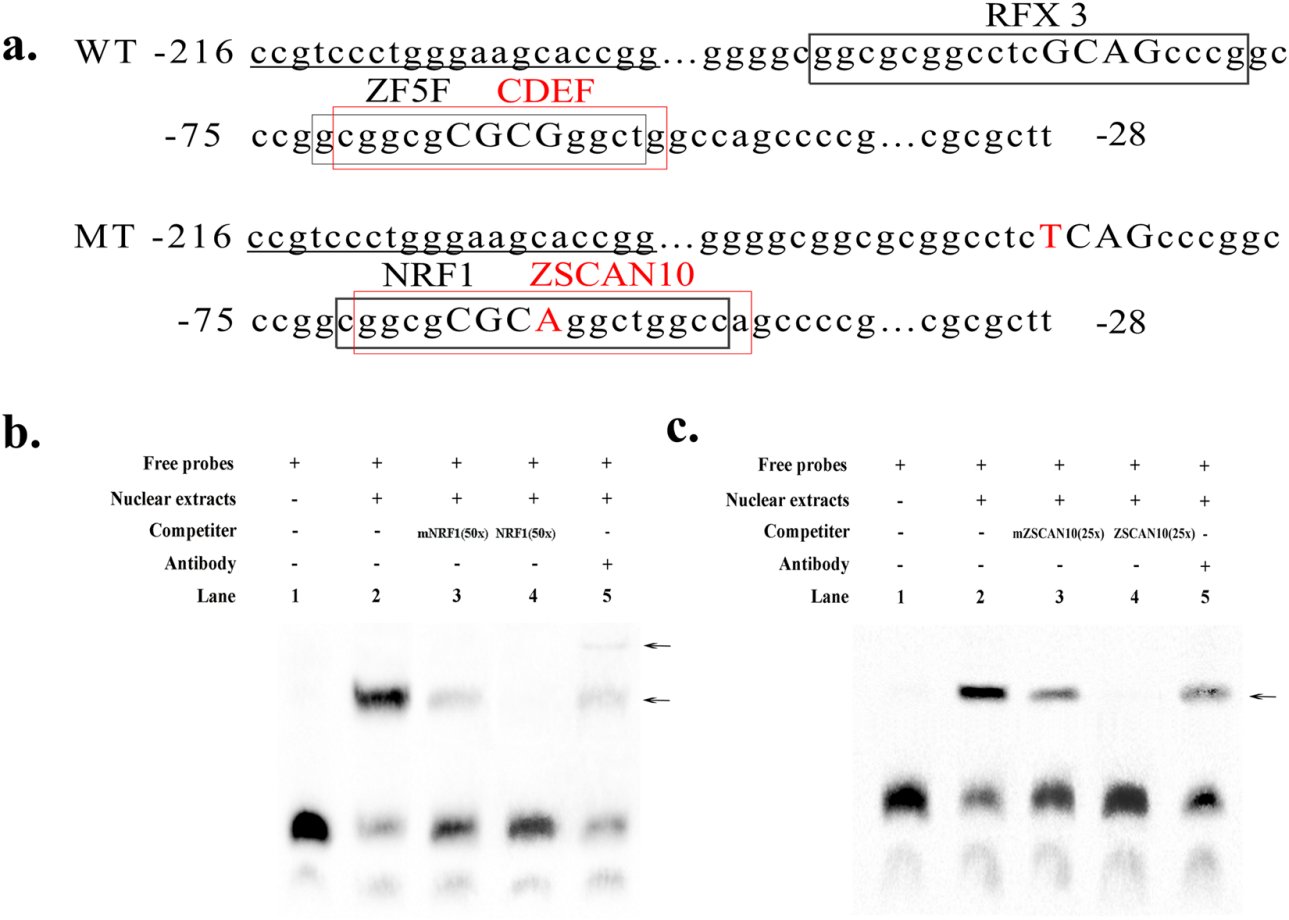
EMSA assays showing direct binding of NRF1 and ZSCAN10 to the SIX1 promoter *in vitro*. (**a**) Sequence and putative transcription factor-binding sites in the wild type (WT) and mutation type (MT) in the proximal minimal promoter of SIX1 gene. The putative transcription factor binding sites are boxed. The primers for the unidirectional deletions are underlined. (**b**) Nuclear protein extracts were incubated with a free probe containing the NRF1 binding site in the presence or absence of any competition (lane 2), 50× mutation probe (lane 3) and 50× unlabelled probe (lane 4). The super-shift assay was conducted using 10 μg anti-NRF1 antibodies (lane 5). (**c**) Nuclear protein extracts were incubated with a free probe containing the ZSCAN10 binding site in the presence or absence of any competition (lane 2), 25× mutation probe (lane 3) and 25× unlabelled probe (lane 4). The super-shift assay was conducted using 10 μg anti-ZSCAN10 antibodies (lane 5). The arrows mark the main complexes.

To detect the transcriptional activities of the haplotypes, the various haplotypes were cloned, and then luciferase reporter, named pGL3-Hap1, pGL3-Hap2, pGL3-Hap3 and pGL3-Hap4, were constructed. The transcriptional activities of these haplotypes were determined using a dual-luciferase reporter assay system in the QCMCs. The results showed that Hap1 had a 0.83-fold (*P* < 0.01), 0.04-fold (*P* > 0.05) and 0.91-fold (*P* < 0.01) lower activity than Hap2, Hap3 and Hap4, respectively (Fig. 4). A pairwise comparison of the transcriptional activities of Hap1 and Hap2 (different at the SNP2 locus, *P*_Hap1/2_ < 0.01), Hap3 and Hap4 (different at the SNP2 locus, *P*_Hap3/4 < _0.01), Hap1 and Hap3 (different at the SNP1 locus, *P*_Hap1/3_ > 0.05), Hap2 and Hap4 (different at the SNP1 locus, *P*_Hap2/4_ > 0.05) was performed. We hypothesized that SNP2 in the g. −63 G > A mutation potentially resulted in the binding of the transcription-factor Nuclear respiratory factor 1 (NRF1) and Zinc finger and SCAN domain containing 10 (ZSCAN10) proteins, which may play a prominent role in the regulation of transcription activities (Table S2, Fig. 5a).

### NRF1 and ZSCAN10 bind to the promoter region of the bovine SIX1 gene *in vitro* and *in vivo*

**Figure 6:**
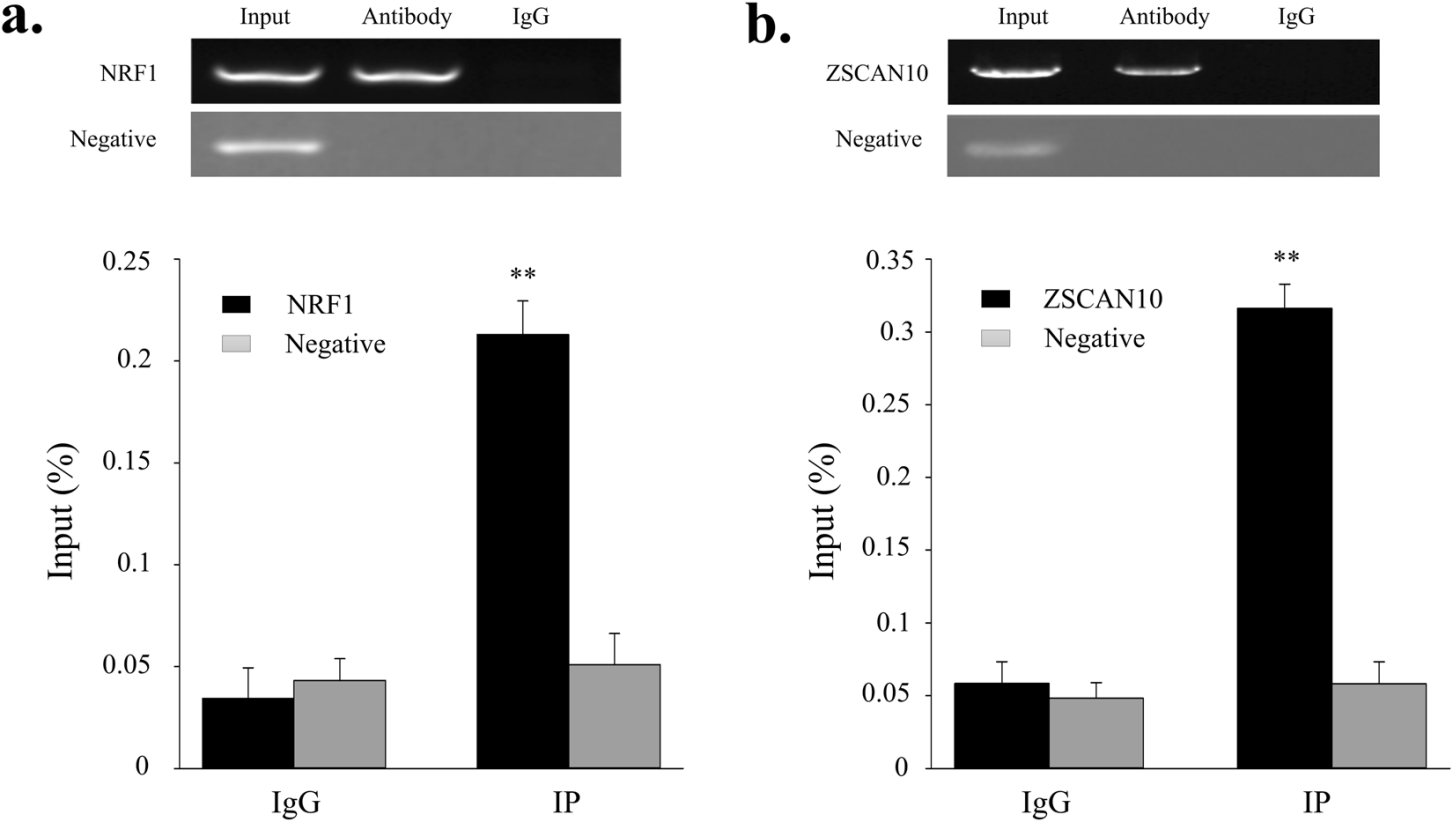
ChIP assay of NRF1 and ZSCAN10 binding to the SIX1 promoter *in vivo*. We analysed the immunoprecipitated products of the NRF1 (**a**) and ZSCAN10 (**b**) antibodies via RT-PCR and ChIP-QPCR. We used total chromatin from muscles as the input, and normal rabbit IgG served as the negative control antibody. “**”*P* < 0.01. Error bars represent the SD (n = 3).

Electrophoretic mobility shift assays (EMSAs) and a chromatin immunoprecipitation assay (ChIP) were used to determine whether NRF1 and ZSCAN10 bind to the promoter region of the bovine SIX1 gene (Fig. 5a), resulting in increased transcriptional activities of Hap2 and Hap4. As shown in Fig. 5b, the nuclear protein from the QCMCs bound to the 5′-biotin labelled NRF1 probes and formed two main complexes (lane 2, Fig. 5b). The competition assays verified that the mutant probe had a slight effect on the main complexes (lane 3 Fig. 5b). However, specificity of the NRF1/DNA interaction was prevented by competition from excess non-labelled DNA (lane 4, Fig. 5b). The final lane shows that the complex was super-shifted when it was incubated with the NRF1-antibody (lane 5, Fig. 5b). ZSCAN10 yielded similar results as NRF1 (Fig. 5c). Although the EMSA in the ZSCAN10 experiments did not reveal a super-shifted product at the ZSCAN10 binding sites, however, the brand of the main complex was clearly decreased. The super-shifted may have formed a high molecular weight polymer, which caused a reduced gel mobility shift (lane 5, Fig. 5c). The ChIP results revealed that NRF1 and ZSCAN10 interacted with the binding sites; the relative enrichment levels were ~4.9 and ~6.6-folds over the IgG control respectively (Fig. 6a and b), respectively, based on three independent experiments.

## Discussion

Identifying QTLs and candidate genes that can be utilized in marker-assisted breeding through the manifestation of economically important traits will facilitate Qinchuan cattle breeding programmes. Variants of candidate genes can be associated with economically important traits, such as growth and carcass traits^18, 19^. SIX1 has emerged as a candidate gene that is an important regulator of vertebrate development and the maintenance of differentiated tissues states^20, 21^. Our work supports that SIX1 is one of the genes that controls myogenesis and may influence BMTs.

In the present study, the tissue distribution of SIX1 mRNA showed the highest expression in the *longissimus thoracis* (Fig. 1a), which is consistent with previous findings observed in other species, such as in human^22^, porcine^23^ and duck^24^. However, the SIX1 expression level is very low in the kidney in this study, which differs from the results of previous studies in which SIX1 was shown to be a crucial factor for early kidney development^6, 7^. The main reason for this discrepancy is that SIX1 expression is regulated temporally in the kidney during early embryonic development^6, 7^. Previous studies have mainly studied the regulatory mechanism of the SIX1 gene in early embryogenesis, particularly in the successive steps of myogenesis, but less is known about its expression pattern and regulatory mechanism during later development. In the present study, we detected an up-regulation of SIX1 expression from 1 to 12 months after birth; however, the expression of SIX1 decreased dramatically after 18 months. Increases in skeletal muscle mass are largely determined by hypertrophy of muscle fibers during postnatal growth because the number and size of muscle fibres do not change after birth^25^. An increase in muscle mass solely through muscle fibres hypertrophy could influence the body measurement and meat quality^26^. Qinchuan cattle require on average of 12 to 24 months attaining physiological and skeletal maturity, which appears closely related to the decreasing SIX1 expression during this time (Fig. 1b and c). Overall, the mechanism of the bovine SIX1 gene may be associated not only with the early events of myogenesis but also with muscle growth and meat quality.

Previous studies have shown that genetic polymorphisms in SIX1 are associated with BMTs. In pigs, Wu *et al*.^23^ reported that a C/T and A/G polymorphism in the SIX1 promoter and intron were significantly associated with the meat color value (MCV1) and meat marbling (MM1) of *longissimus dorsi* and dressing percentage (DP). In the present study, the correlation analysis showed that cattle with the G allele at g. −85 G > T and the A allele at g. −63 G > A loci had better BMTs. A pairwise comparison revealed that a SNP2 mutation at g. −63 G > A associated with the prominent role of transcription-factors NRF1 and ZSCAN10 in the proximal minimal promoter region, which play a major role in transcriptional regulation^27, 28^, thus contributing to regulate the transcriptional activity of the SIX1 gene.

NRF1, which encodes a protein that homodimerizes and functions as a transcription factor, is associated with neurite outgrowth through the regulation of globin gene expression^29^. Previous studies have shown that the activation of NRF1 in fibroblasts induces increases in cytochrome *c* expression and mitochondrial respiratory capacity. Overexpressing NRF1 in skeletal muscle resulted in an increased expression of myocyte enhancer factor (MEF) 2 A and GLUT4^30^, which were associated with a proportional increase in insulin-stimulated glucose transport in muscles^31^. Moreover, NRF1 can interact with DYNLL1^32^, PPARGC1A^33^ and Cytochrome *c* oxidase (COX)^34^, to initiate nitric oxide synthase^33^ and active energy metabolism^34^ for individual development. ZSCAN10 (also named as Zfp206) belongs to the C2H2 zinc fingers family and encodes a zinc finger transcription factor that is specifically expressed in embryonic stem cells (ESCs)^35, 36^. ZSCAN10 is a regulator of pluripotency and maintains the pluripotent state by interacting with other pluripotency factors, such as Sox2 and Oct4^37^. ZSCAN10-null mice have a reduced weight and mild hypoplasia in the heart, spleen, eyes and long bones^38^. Consistently, ZSCAN10 expression in ESCs mid-gestation embryos and adults suggests that ZSCAN10 plays a role in maintaining progenitor cell subpopulation and regulating individual development. In the present study, we identified that NRF1 and ZSCAN10 binds to Hap2 and Hap4 and induce a significantly higher transcriptional activity than others based on the luciferase reporter, EMSA and ChIP assays. These results demonstrate that NRF1 and ZSCAN10 play important roles in regulating the transcriptional activity of the SIX1 gene and contributing to better body measurements.

In combination, the H_1_-H_2_ and H_3_-H_4_ diplotypes showed better BMTs than the H_1_-H_1_, H_1_-H_3_ and H_3_-H_3_ diplotypes. Correlation analysis results were consistent with the interpretation that the transcriptional activities of Hap2 had a higher activity than those Hap1 and Hap3. Notably, the H_3_-H_4_ diplotypes showed a lower IFC than the other diplotypes, which is an indicator of lean meat quality characteristics, affects juiciness, flavour, and morphology and is closely related to beef tenderness^15^. There is significant breed variation in IFC, with the Belgian Blue bulls producing the leanest meat, Limousine producing intermediate levels and Aberdeen Angus producing the highest fat content^39, 40^. This increased IFC has a positive impact on tenderness, taste and flavour^40^. Through aggregate selection, based on our findings, we infer that the H_1_-H_2_ diplotypes could be used as a molecular marker of combined genotypes for future selection of BMTs in Qinchuan cattle.

In conclusion, our study revealed that bovine SIX1 is highly expressed in the *longissimus thoracis* and physiological immature individuals. Two SNPs were located within the proximal minimal promoter region from −216 to −28, inducing the binding of transcription factors NRF1 and ZSCAN10 on the promoter region of diplotypes H_1_-H_2_ and regulating SIX1 transcriptional activity. Correlation analysis showed the combined haplotypes H_1_-H_2_ (-GG-GA-) had significantly greater BMTs than the others. This information may be useful for MAS in cattle breeding.

## Materials and Methods

### Ethics Statement

All animal procedures were performed according to guidelines laid down by the China Council on Animal Care, and the protocols were approved by the Experimental Animal Manage Committee (EAMC) of Northwest A&F University.

### Animal sources, data collection, and genomic DNA isolation

In total, 428 female cows aged 18 to 24 months were randomly selected from the National Beef Cattle Improvement Center’s experimental farm (Yangling, China). The BMTs were measured as described previously^41^, including BL, WH, CD and CC. ULA, BF and IFC were measured via ultrasound using a Sono-grader model 2 (Renco, USA). Genomic DNA was extracted from blood samples, using standard method^42^, and stored at −20 °C until subsequent analyses.

### Real-time PCR analysis of expression pattern

Fifteen tissues (heart, liver, spleen, kidney, rumen, reticulum, omasum, abomasa, small intestine, large intestine, subcutaneous fat, *longissimus thoracis*, soleus, psoas and testicle) were obtained from three adult Qinchuan cattle. The *longissimus thoracis* samples were collected during eight developmental stages from male Qinchuan cattle, including 1, 3, 6, 12, 18, 24, 36 and 48 months after birth, and three parallel individuals were sampled during each period. Total RNA was extracted from the tissues using a Total RNA Kit (Tiangen, Beijing, China) and then reverse-transcribed using a PrimeScript™ RT Reagent Kit (Perfect Real Time) (TaKaRa, Dalian, China). The reaction was performed using a SYBR Green PCR Master Mix Kit (TaKaRa) on a 7500 System SDS V 1.4.0 (Applied Biosystems, USA). All primers used in the real-time PCR experiments are listed in Supplementary Table S1. Glyceraldehyde-3-phosphate dehydrogenase (GAPDH) was invoked as the endogenous control gene. The relative expression levels of the target mRNAs were calculated using the 2^−ΔΔCt^ method^43^.

### Western blotting

Tissues protein were extracted using the T-PER Tissue Protein Extraction Reagent (Pierce, Thermo Fisher Scientific, USA). The total protein samples were quantified using the Pierce BCA Protein Assay Kit (Thermo Scientific), and 50 μg were electrophoresed on a 10% SDS-polyacrylamide gel, and then transferred to nitrocellulose. After blocking in defatted milk powder, the membranes were incubated with the SIX1 antibody (sc-514441, Santa Cruz, USA) and GAPDH antibody (sc-293335, Santa Cruz). The blots were washed and subsequently treated with a peroxidase labelled secondary antibody. The signals were detected as chemical luminescence to expose X-ray films using the ChemiDoc™ XRS+ System (Bio-Rad, Hercules, CA, USA).

### 5′-Rapid amplification of cDNA ends (5′-RACE)

To identify the transcriptional start site (TSS) of the bovine SIX1 gene, 5’-RACE was performed on total RNA from the *longissimus thoracis* muscle using a BD SMARTTM RACE cDNA amplification kit (Clontech Inc., CA, USA) according to the manufacturer’s protocol. PCR was performed using a Universal Primer A Mix (UPM, Clontech Inc., CA, USA) and the nested PCR primers (Table S1) located in exon 1 of the SIX1 gene. The conditions and methods used were as previously described^27^. For sequencing PCR products were separated by electrophoresis in 2% agarose gels and subsequently cloned into T-Vector pMD19 (simple) (Takara).

### Primer design, PCR amplification, and SNPs detection

Three pairs of PCR primers (primer A, B and C) were designed to amplify a 1.8 kb genomic region upstream of the bovine SIX1 gene (AC_000167.1 from 73068130 to 73074697). The primer sequences are reported in Table S1. The PCR amplifications were carried out using pooled genomic DNA from 428 Qinchuan cattle as a template^44^. The 20 μL PCR reaction volume contained 50 ng of pooled genomic DNA, 0.5 μM of primer, 1 × buffer (including 1.2 mM MgCl_2_), 200 μM dNTPs, and 0.4 units of KOD DNA polymerase (Toyobo, Osaka, Japan). The PCR was performed using a program of 5 min at 95 °C, 34 cycles of 97 °C 30 s, an annealing temperature for the primers as shown in Table S1 for 30 s and 72 °C for 60 s. All PCR products were sequenced to verify amplification of the intended target. Finally, the sequences were imported into BioXM software (Version 2.6) for the SNP analysis.

### Promoter cloning and generation of the luciferase reporter constructs

Fragment primers for −1802, −1488, −1044, −708, −483, −216, −28 and +144 were designed (Table S1) to amplify unidirectional deletions of the bovine SIX1 promoter. Promoter constructs were generated by PCR using specific primers with the sequence of the *Kpn*I and *Bgl*II restriction sites incorporated and the wild individuals as DNA templates. Then, all fragments were cloned into Vector pMD19-T (simple) (TaKaRa), and ligated into the luciferase reporter construct pGL3-basic vector digested with the same restriction enzymes *Kpn*I and *Bgl*II (TaKaRa). These plasmids were named pGL3-1802, pGL3-1488, pGL3-1044, pGL3-708, pGL3-483, pGL3-216 and pGL3-28. The PCR amplification conditions were similar to the conditions used in the previous step and plasmid DNA was further confirmed by DNA sequencing.

### Potential *cis*-acting elements identification

The Genomatix database (http://www.genomatix.de) was used to search for potential *cis*-acting elements in the proximal minimal promoter region of the SIX1 gene. These potential *cis*-acting elements were compared for genotypic differences. Sequences that contained one or two SNPs or being adjacent to SNPs were evaluated.

### Cell culture and transfection

QCMCs were isolated from Qinchuan foetal bovine as described previously^45, 46^. The QCMCs were maintained in Dulbecco’s Modified Eagle Medium (DMEM-F12) and supplemented with 20% newborn calf serum (NBCS, Invitrogen, USA) and antibiotics (100 IU/mL penicillin; 100 µg/mL streptomycin) at 37 °C and 5% CO_2_ in a normal atmosphere incubator. Cells were grown overnight to 80–90% confluence at a density of 1.2 × 10^5^ cells in the growth medium without antibiotics in 24-well plates. In each well, 800 ng of the each series of reporter plasmid (pGL3 −1802, −1488, −1044, −708, −483, −216 and −28) were co-transfected with pRL-TK normalizing reporter plasmid (Promega, USA) into QCMCs with 3 μL X-tremeGENE HP DNA transfection reagent (Roche, USA). The pGL3-Basic vector served as a negative control. At 5 h after the transfection, we replaced the media with DMEM with 2% horse serum (HS) (GIBCO, Invitrogen) and incubated for 40 h to induce the differentiation of the QCMC myoblasts into myotubes. We performed all remaining steps as previously described^28^. Firefly luciferase activity and Renilla luciferase activity were measured according to the dual-luciferase reporter assay standard protocol in three independent experiments. The relative luciferase activities were determined using a NanoQuant Plate™ (TECAN, infinite M200PRO).

### Statistical analysis

Statistical analysis was performed for allelic frequencies, genotype frequencies, *He*, Hardy-Weinberg equilibriums and *PIC* parameters according to Nei’s methods^17^. Linkage Disequilibrium (LD) and haplotype distributions of the SNPs were analyzed using the expectation maximization algorithm with Haploview software^47^. The association between single SNPs and body measurements traits were analyzed using the general linear models (GLM) procedure in SPSS (version 13.0). The linear model was used:

$${{\rm{Y}}}_{{\rm{ijkl}}}={\rm{u}}+{{\rm{G}}}_{{\rm{i}}}+{{\rm{A}}}_{{\rm{j}}}+{{\rm{A}}}_{{\rm{k}}}+{{\rm{S}}}_{{\rm{l}}}+{{\rm{E}}}_{{\rm{ijkl}}}$$

where Y_ijkl_ were the traits measured on each individual cow, μ was the overall population mean for the traits, G_i_ was the fixed genotype effect, A_j_ was the fixed effect of age, A_k_ was the fixed effect due to the age of dam, S_l_ was the fixed effect due to the season of sampling (spring vs. fall) and E_ijkl_ was the standard error.

### EMSA assays

Nuclear extracts from the QCMCs were prepared using the Nuclear Extract Kit (Active Motif Corp., Carlsbad, CA, USA) according to the manufacturer’s protocol. The Bradford dye assay (Bio-Rad Corp., Richmond, CA, USA) was used to adjust the concentration of nuclear fraction protein. The LightShift Chemiluminescent EMSA Kit (Thermo Fisher Corp., Waltham, MA, USA) was used for the EMSA assays according to the manufacturer’s protocol. Briefly, 200 fmol of the 5′ end with biotin labelled probes (listed in Table S1) were incubated room temperature for 20 min with a reaction mixture containing 2 μL 10 × binding buffer, 1 μL poly (dI^.^dC) and 10 μg of nuclear protein extract in a volume of 20 μL. For the competition assay, unlabelled or mutated DNA probes were added to the reaction mixture and incubated for 15 min; then, 200 fmol labeled probes in a volume of 20 μL were added and incubated at room temperature for 20 min. For the super-shift assay, 10 μg of the NRF1 (ab86516, Abcam, USA) or ZSCAN10 (ab45344, Abcam) antibodies were added to the reaction mixture and then incubated on ice for 30 min; then, 200 fmol of the labelled probes in a volume of 20 μL were added and incubated at room temperature for 20 min. Finally, the main complexes were resolved on 6% non-denaturing polyacrylamide gel electrophoresis (PAGE) using 0.5 × TBE buffer for 1 h and image with ChemiDoc™ XRS+ System molecular imager (Bio-Rad).

### ChIP assay

The ChIP assays were performed using the SimpleChIP® Enzymatic Chromatin IP Kit (CST, Massachusetts, USA) according to the manufacturer’s protocol. The samples (n = 3) from the Hap2 and Hap4 Qinchuan bovine were used. The protein-DNA complexes were cross-linked with 37% formaldehyde and neutralized with glycine. After digesting the DNA with micrococcal nuclease into fragments of approximately 150–900 bp in length, the fragmented chromatin samples were suspended in the ChIP dilution buffer. The cross-linked chromatin samples were immunoprecipitated with 4 μg of the NRF1 or ZSCAN10 antibodies and normal rabbit IgG overnight at 4 °C. The immunoprecipitated products were isolated with protein G agarose beads, and the bound chromatin was then collected with salt washes. The eluted ChIP in the Elution Buffer was then digested with proteinase K and purified for a quantitative PCR analysis. The ChIP primers used in the RT-PCR experiment are listed in Table S1. Percent input was calculated as follows: % Input = 2^[−ΔCt(Ct[ChIP] − (Ct[Input]−Log2(Input Dilution Factor)))]^^48^. We used the immunoprecipitated products from the normal rabbit IgG group as a negative control.

## Electronic supplementary material

SUPPLEMENTARY INFO
