# Author Correction: NRF1 and ZSCAN10 bind to the promoter region of the SIX1 gene and their effects body measurements in Qinchuan cattle

**DOI:** 10.1038/s41598-022-12159-8

**Published:** 2022-05-13

**Authors:** Da-Wei Wei, Lin-Sheng Gui, Sayed Haidar Abbas Raza, Song Zhang, Rajwali Khan, Li Wang, Hong-Fang Guo, Lin-Sen Zan

**Affiliations:** 1 College of Animal Science and Technology, Northwest A&F University, Yangling, 712100 Shaanxi People’s Republic of China; 2 National Beef Cattle Improvement Center, Northwest A&F University, Yangling, 712100 Shaanxi People’s Republic of China; 3 Modern Cattle Biotechnology and Application of National-Local Engineering Research Center, Yangling, 712100 Shaanxi People’s Republic of China; 4 Shaanxi Beef Cattle Engineering Research Center, Yangling, 712100 Shaanxi People’s Republic of China

Correction to: *Scientific Reports* 10.1038/s41598-017-08384-1, published online 11 August 2017

The original version of this Article contains an error in Figure 1, where the SIX1 and GAPDH are incorrect in panel (c). The correct Figure 1 and accompanying legend appear below.

**Figure 1:**
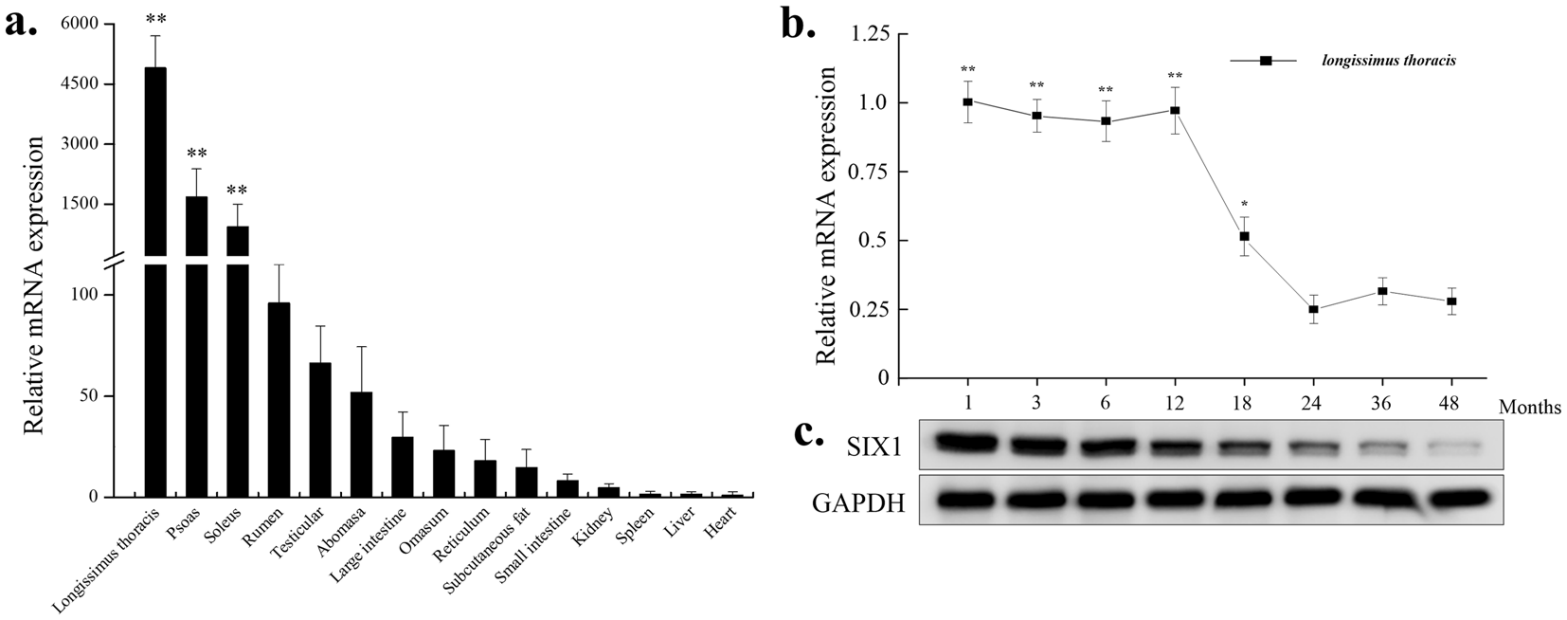
Expression pattern analysis of the bovine SIX1. (**a**) Analysis of the bovine SIX1 expression pattern in tissues and organs. (**b**) Expression pattern of the bovine SIX1 mRNA at different developmental stages. (**c**) Bovine Six1 expression pattern at different developmental stages. The samples of the *longissimus thoracis* were obtained at 1, 3, 6, 12, 18, 24, 36 and 48 months after birth. SIX1 mRNA expression was normalized to the housekeeping gene GAPDH and the expression levels were calculated relative to the gene expression in the liver and 24 months, respectively. The value of each column represents the mean ± standard deviation of three independent experiments. The unpaired Student’s t-test was used to detect significant differences. “*”*P* < 0.05 and “**”*P* < 0.01.

