# Supplementary material for: NRF1 and ZSCAN10 bind to the promoter region of the SIX1 gene and their effects body measurements in Qinchuan cattle
Source: Sci Rep. 2017 Aug 11;7:7867. doi: 10.1038/s41598-017-08384-1 (PMC5554236; doi:10.1038/s41598-017-08384-1)
Supplement: Supplementary file 1 — SUPPLEMENTARY INFO [file 41598_2017_8384_MOESM1_ESM.pdf]

NRF1 and ZSCAN10 bind to the promoter region of the SIX1 gene and their effects body measurements in Qinchuan cattle

Da-Wei Wei[1,4†], Lin-Sheng Gui[1,4†], Song-Zhang[1,4], Rajwali Khan[1], Sayed Haidar Abbas Raza[1], Li-Wang[1,4], Hong-Fang Guo[1,4] and Lin-Sen Zan[1,2,3]

[1]College of Animal Science and Technology, Northwest A&F University, Yangling 712100 Shaanxi, People's Republic of China. [2]National Beef Cattle Improvement Center, Northwest A&F University, Yangling 712100 Shaanxi, People's Republic of China. [3]Modern Cattle Biotechnology and Application of National-Local Engineering Research Center, Yangling 712100 Shaanxi, People's Republic of China. [4]Shaanxi Beef Cattle Engineering Research Center, Yangling 712100 Shaanxi, People's Republic of China. Correspondence and requests for materials should be addressed to L.-S.Z. (email: zanlinsen@163.com).

[†] These authors contributed equally to this study.

Table S1. Primers used in the expression and functional analysis of the SIX1 promoter experiments

Reaction	Name	Function	Primer Sequence (5' to 3')	Tm (°C)	Product Length(bp)	Amplified Region
qRT-PCR	GAPDH	Reference	F: CCAACGTGTCTGTTGTGGAT<br>R: CTGCTTCACCACCTTCTTGA	61.0	80	778-857
	SIX 1	RT-PCR	F: GCCAAGGAAAGGGAGAACA<br>R: GACTCTGGGGAGGTGAGAACT	61.0	127	866-992
5' RACE	R1		GGCAGGGTCTACCAAGCACCGTTTCC	66.5	555	
	R2		CACTCCGCTCTTGACCACCGAGA	60.0	475	
Promoter cloning	Primer A	5'flanking region	F: ATCAGCATTTGCTTCCAAGT<br>R: TTGAGAGATGTCTTGCAATTTC	62.5	728	-1824/-1097
	Primer B	5'flanking region	F: ACTAAGCATTCTCCACACATAC<br>R: TGTTGGAATGAATAAGTAATG	59.0	750	-1164/-415
	Primer C	5'flanking region	F: GTCTACGCTTGTATTATTTATCG<br>R: AAGGAACGGCCGGCTCACTC	61.5	602	-483/+118
	-1802	5'flanking region	F: *CGGGGTACC*TAATTGCCTGGGATACTGAA	63.5	1947	-1802/+144
	-1488	5'flanking region	F: *CGGGGTACC*GAAGCAAATTTAAAGAACCTA	62.0	1633	-1488/+144
	-1044	5'flanking region	F: *CGGGGTACC*CACACACATGCACACACACAC	65.0	1189	-1044/+144
	-708	5'flanking region	F: *CGGGGTACC*AATCTCTATTTGCTCAGCAAC	60.5	853	-708/+144
	-483	5'flanking region	F: *CGGGGTACC*GTCTACGCTTGTATTATTTATCG	64.0	628	-483/+144
	-216	5'flanking region	F: *CGGGGTACC*CTCACGTTGCAAGGTCCTGAC	63.5	361	-216/+144
	-28	5'flanking region	F: *CGGGGTACC*CAATCAGCGCGCCGAGAACGC	60.0	173	-28/+144
	+144	Exon 1	R: *GGAAGATCT*GAGGAGGAGTAGAGCGCGCGC			
EMSA	NRF1 forward		CCGGCCCGGCGGCG***CGCA***GGCTGGCCAGCCCCGG			-80/-46
	NRF1 reverse		CCGGGGCTGGCCAGCC***TGCG***CGCCGCCGGGCCGG			
	mNRF1 forward		CCGGCCCGGCGGCG***TTTT***GGCTGGCCAGCCCCGG			-80/-46
	mNRF1 reverse		CCGGGGCTGGCCAGCC***AAAA***CGCCGCCGGGCCGG			
	ZF15 forward		CGGCCCGGCGGCG***CGCA***GGCTGGCCAGCCCCGGA			-79/-45
	ZF15 reverse		TCCGGGGCTGGCCAGCC***TGCG***CGCCGCCGGGCCG			
	mZF15forward		CGGCCCGGCGGCG***TTTT***GGCTGGCCAGCCCCGGA			-79/-45
	mZF15 reverse		TCCGGGGCTGGCCAGCC***AAAA***CGCCGCCGGGCCG			
ChIP	ChIP-NRF1/ ZSCAN10		F: CCCCACTTCGCTTCTCACCACTC<br>R:CCGGCTCACTCCGCAGCTTTT	60.0	261	-152/+109
	ChIP- control	Exon 2	F: TCCTAAGAAGGGAGGGACTGG<br>R: AGGCTGGGTAACTGGATGTTT	60.0	283	3180/3463

The italicized letters of the forward (F) and reverse (R) primers were enzyme cutting sites of *Kpn*I and *Bgl*II, the underlined bases are core putative transcription factor-binding sites, respectively.

Table S2. The different potential *cis*-acting elements between wild types and SNPs sequence in the proximal minimal promoter of bovine SIX1.

Item	Transcription factors	Optimized	*Cis*-acting elements (Recognition sequence)	Target strand	SNPs
WT1	Regulatory factor X 3 (RFX 3)	0.84	cgggCTGCgaggccgcgcc	(-)	
SNP1	NONE	0.9	cgggCTGAgaggccgcgcc	(-)	g. -85 G > T
WT2	ZF5 POZ domain zinc finger (ZF5F)	0.83	cagccCGCGcgccgc	(-)	
	Cell cycle-dependent element (CDEF)	0.87	ggcgCGCGggctg	(+)	
SNP2	Nuclear respiratory factor 1 (NRF1)	0.92	cggcgCGCAggctggcc	(+)	g. -63 G > A
	Zinc finger and SCAN domain containing 10 (ZSCAN10)	0.86	tggccagccTGCGcgcc	(-)	g. -63 G > A

WT stands for wild type. SNP loci are underlined in the table; capital letters are the core sequence of the transcription factors, and the value > 80 denotes a sequence combination with the *Cis*-acting elements.
